# Enhancement of β-carotene content in *Chlamydomonas reinhardtii* by expressing bacterium-driven lycopene β-cyclase

**DOI:** 10.1186/s13068-023-02377-1

**Published:** 2023-08-12

**Authors:** Danqiong Huang, Chenglong Liu, Mingshan Su, Zhiyong Zeng, Chaogang Wang, Zhangli Hu, Sulin Lou, Hui Li

**Affiliations:** 1 Guangdong Engineering Research Center for Marine Algal Biotechnology, Guangdong Provincial Key Laboratory for Plant Epigenetics, Shenzhen Engineering Laboratory for Marine Algal Biotechnology, Longhua Innovation Institute for Biotechnology, College of Life Sciences and Oceanography, Shenzhen University, Shenzhen, China; 2 Key Laboratory of Optoelectronic Devices and Systems of Ministry of Education and Guangdong Province, College of Physics and Optoelectronic Engineering, Shenzhen University, Shenzhen, China

**Keywords:** β-Carotene, *Chlamydomonas reinhardtii*, Lycopene β-cyclase, Prokaryotic gene, Eukaryotic microalgae, Cell factory

## Abstract

β-Carotene is one of the economically important carotenoids, having functions as the antioxidant to remove harmful free radicals and as the precursor for vitamin A and other high-valued xanthophyll such as zeaxanthin and astaxanthin. Lycopene cyclase plays an important role in the branching of β-carotene and α-carotene. Aiming to develop the microalgae with enhanced β-carotene productivity, the *CrtY* gene from bacterium *Pantoea agglomerans* was integrated into *Chlamydomonas reinhardtii*. The lycopene-producing *E. coli* harboring *CrtY* gene produced 1.59 times of β-carotene than that harboring *DsLcyb1* from *Dunaliella salina* (a microalga with abundant β-carotene), confirming the superior activity of *CrtY* on β-carotene biosynthesis. According to the pigment analysis by HPLC, in microalgal transformants that were confirmed by molecular analysis, the expression of *CrtY* significantly increased β-carotene content from 12.48 mg/g to 30.65 mg/g (dry weight), which is about 2.45-fold changes. It is noted that three out of five transformants have statistically significant higher amount of lutein, even though the increment was 20% in maximum. Besides, no growth defect was observed in the transformants. This is the first report of functional expression of prokaryotic gene in eukaryotic microalgae, which will widen the gene pool targeting carotenoids biosynthesis using microalgae as the factory and thereby provide more opportunity for high-valued products engineering in microalgae.

**Supplementary Information:**

The online version contains supplementary material available at 10.1186/s13068-023-02377-1.

## Introduction

Carotenoids are a group of important natural pigments commonly synthesized by plants, fungi, bacteria, and algae, presenting yellow, orange, red, and even purple colors [1]. Structurally, most carotenoids are terpenoid molecules with C40 backbone driven from the connection of eight isoprene units end-to-end and varied end groups [1–3]. According to the modification of end groups, carotenoids can be divided into two major groups, including carotenes (such as α-carotene, β-carotene, γ-carotene, and lycopene) and xanthophylls (such as β-cryptoxanthin, zeaxanthin, astaxanthin, and lutein) [2, 3]. Currently, there are more than 850 carotenoids were found naturally, including about 50 carotenes and about 800 xanthophylls (up until 2018) [3]. As the major precursor for vitamin A synthesis in the body, people and animals should uptake carotenoids from food to maintain healthy [3]. In photosynthetic organisms, carotenoids play important role in light harvesting for normal photosynthesis [3–5]. Besides, as a chain breaking anti-oxidants, carotenoids could protect cells and organisms against the photo-oxidation releasing harmful free radicals [4–6]. It has been reviewed that carotenoids can be used as medicine for their effective in immune regulation, anti-cancer, and anti-aging [7]. Therefore, carotenoids have a great economic value as nutrients, additives, or medicine in food, feed, aquaculture, pharmaceutical, and cosmetic industries.

**Fig. 1:**
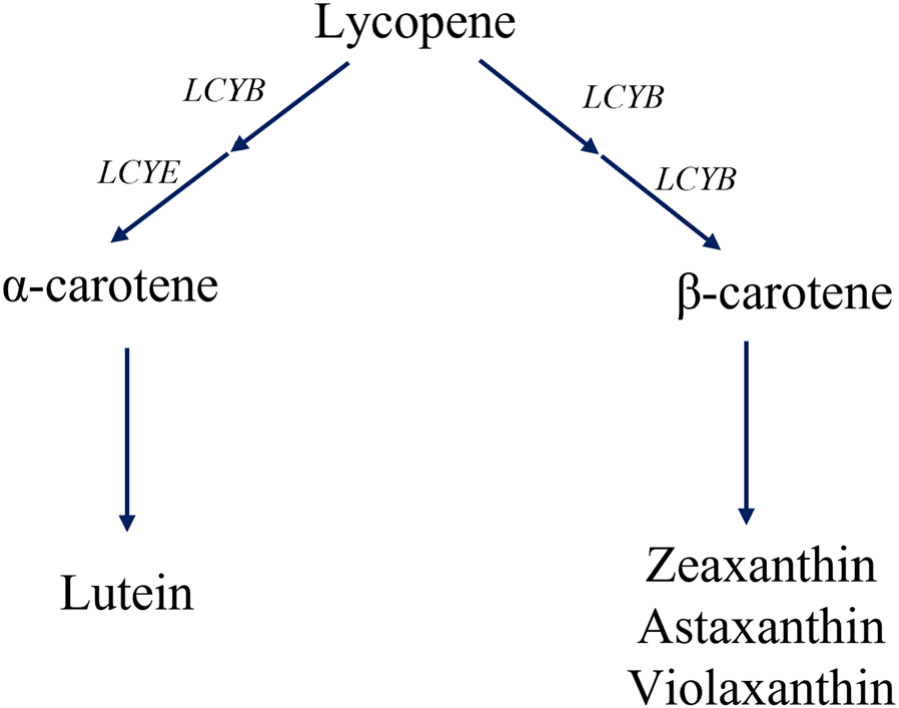
The simple schematic representation of the β-carotene biosynthesis and its competition branch. LCYB adds the β-ring at the end of lycopene while LCYE adds the ε-ring at the end of lycopene

Lots of effects have been placed to achieve carotenoids with high quality and low cost, especially by metabolic engineering approaches, on the basis of well-studied carotenoids biosynthesis pathway [8–11]. It has been noted that astaxanthin, β-carotene, and lutein approximately account 60% of the carotenoids market [12]. In the other hand, β-carotene is an important node in the carotenoid metabolism, because it is the precursor of lots of xanthophylls such as β-cryptoxanthin, zeaxanthin and astaxanthin, as well as it is also the competitor of other xanthophylls such as lutein [7, 8]. Therefore, microbial cell factories such as *Escherichia coli*, yeast, and microalgae were engineered to improve its productivity, through the integration of key enzymes or the whole gene cluster associated with carotenoids biosynthesis [13–18]. According to the documentation, the type of lycopene cyclization leads to the branch division of α-carotene and β-carotene [8] (Fig. 1). Previously, four major groups of lycopene cyclase have been identified, including (1) the CrtY-type β-cyclase from many carotenogenic proteobacteria, *Streptomyces* spp., and the *Chloroflexi*; (2) CrtL-type cyclase in some cyanobacteria and plants; (3) the heterodimeric cyclase from some Gram-positive bacteria; and (4) CruA/CruP-type cyclase in green sulfur bacteria and cyanobacterial genomes that lack the CrtL-type lycopene cyclase [19–21]. The *CrtYB* from red yeast *Xanthophyllomyces dendrorhous,* belonging to the heterodimeric cyclase group and displaying biofunction of phytoene synthase and lycopene cyclization, has been expressed in microalgae *Chlamydomonas reinhardtii* and increased the β-carotene content by 72% [16]. Besides, the overexpression of native LCYE gene in *C. reinhardtii* significantly increased total lutein production at the maximum of 2.6-fold than the wild type and did not significantly increase the β-carotene content [22]. However, even though CrtY-type β-cyclase has been widely used for the β-carotene production in *E. coli*, *Saccharomyces cerevisiae*, *Y. lipolytica*, and *Rhodobacter sphaeroides*, its performance on microalgae targeting on the increased β-carotene productivity has not been studied [23].

In this study, the CrtY from bacterium *Pantoea agglomerans* belong to CrtY-type β-cyclase was tested in *E. coli* to confirm its superior performance on β-carotene production, compared with alga-driven lycopene β-cyclase. Subsequently, *CrtY* was integrated into the genome of *C. reinhardtii* and functionally expressed. This is an example of successful expression of prokaryotic gene in eukaryotic microalgae, which will widen the gene pool targeting carotenoids biosynthesis and thereby provide more opportunity for high-valued products engineering in microalgae.

## Materials and methods

### Strains, culture conditions, and sampling

A cell wall-deficient microalgae strain, *Chlamydomonas reinhardtii* cc849, used in this study was obtained from Guangdong Technology Research Center for Marine Algal Bioengineering. The algal cells of cc849 were cultured in Tris–acetate–phosphate (TAP) medium under a photoperiod of 16/8 h light and dark cycle with the photon fluence rate of 50 µmol m^−2^ s^−1^ at 22 °C in a growth chamber. Transformed algal cells were maintained in the TAP plate containing 8 μg/mL zeocin and 100 μg/mL ampicillin and proliferated in the TAP liquid medium without antibiotics. For the high light treatment, algal cells at the late of logarithmic phase were exposed to a photon fluence rate of 900 µmol m^−2^ s^−1^ for 6 h.

The *E. coli* strain DH5α was used to proliferate the constructed plasmid and the *E. coli* strain BL21(DE)3 was used to produce carotenoids in this study. The lycopene-producing BL21(DE)3 was maintained previously in our lab, containing the plasmid pAC-Lyco, which containing the gene cluster producing lycopene including CrtE, CrtI, CrtB, idi and CrtY genes adapted from pFZ153 [24] (Additional file 1: Fig. S1A) with chloramphenicol selection marker. Both DH5α and BL21(DE)3 cells were cultured in LB media with or without proper antibiotics (100 μg/mL ampicillin and/or 34 ug/mL chloramphenicol) at 37 °C in an incubator with or without shaking at 220 rpm. For the production of carotenoids, IPTG was added into the culture. Algal cells and *E. coli* cells were harvested by centrifugation at 4000 rpm for 8 min at 4 °C and then frozen by liquid nitrogen for further processing.

### Plasmid construction

To construct the plasmid expressing lycopene β-cyclase in *E. coli*, the vector pET-Duet-1 was used as the backbone. Lycopene β-cyclase genes used for plasmid construction included *CrtY* from *Pantoea agglomerans* (GenBank accession number: WP_062759152) and *DsLcyb1* from *Dunaliella salina* (GenBank accession number: ACA34344). After codon-optimization based on *E. coli*, *DsLcyb1* was synthesized and cloned into pET-Duet-1 at *BamHI*/*EcoRI* site, to construct pET-DsLcyb1. The *CrtY* was amplified from pFZ153 [24] with primers CrtYF20 (5′-GCCAggatccATGCCGCGGTATGATCTGATTC-3′) and CrtYR20 (5′- GCTCgaattcATAGTAATCCTCCTTCATTGCATC-3′) containing *BamHI* and *EcoRI*, respectively. PCR was performed using SuperFi DNA polymerase (Invitrogen Life technologies, Carlsbad, CA, USA) as recommended. The PCR products of *CrtY* were digested with *BamHI*/*EcoRI* and then subcloned into pET-Duet-1 to form pET-CrtY. The restriction enzyme digestion (Thermo Scientific FastDigest) and vector construction using T4 DNA ligase (Invitrogen) were performed as the manual.

To construct the plasmid expressing lycopene β-cyclase in microalga *C. reinhardtii*, the vector pDb124 was used, which contains the psaD promoter and psaD terminator to drive the expression of target gene (Additional file 1: Fig. S1B). pDb124 also contains the bleomycin reporter cluster that can be used to screen transformants. *CrtY* was codon-optimized for better expression according to the nucleotide preferences of *C. reinhardtii* nuclear genome. Additionally, a chloroplast signal peptide (CTP) was fused to the N-terminal of *CrtY*. The codon-optimized *CTP*-*CrtY* was synthesized and cloned into the pDb124 at the *NheI* site to form pDb-CrtY*.* The synthesis and subclone of target nucleotides were performed by GenScript Biotech Corp. (Nanjing, China). The accuracy of nucleotides in each plasmid was confirmed by sequencing.

### *E. coli* and algal transformation

To produce target carotenoids in *E. coli*, plasmids pET-DsLcyb1 and pET-CrtY were transferred into the chemical competent cell of lycopene-producing *E. coli* using heat-shock method [25]. To produce target carotenoids in *C. reinhardtii*, the plasmid pDb-CrtY was transferred into algal cells using glass-bead method with few modifications [26]. The algal transformation process was performed as described, with additional information that *NotI* was used to linearize plasmids.

### DNA/RNA extraction and PCR/qPCR analysis

The proliferated plasmid DNA was extracted from *E. coli* strain DH5α using Omega Plasmid Mini Kit II (Omega Bio-Tek, USA). Genomic DNA was extracted from algal cells using M5 HiPer Plant Genomic DNA Kit (Mei5 Biotechnology, Beijing, China) and total RNA was extracted using SteadyPure Plant RNA Extraction Kit (Accurate Biotech. Co., Ltd, Hunan, China). The first strand of cDNA was synthesized by PrimeScriptTM RT reagent Kit with gDNA Eraser (Takara, Dalian, China). The DNA/RNA extraction and reverse transcription using commercial kits were performed according to the corresponding manual.

For the PCR using genomic DNA to evaluate the presence of target genes in transformants, a pair of primer psaD-P (5′—GGGAATTGGAGGTACGACCGAGAT-3′) and psaD-T (5′—AGCTCCGATCCCGTATCAATCAGC-3′) was used, which locating at the psaD promoter and psaD terminator region, respectively. PCR was accomplished using 2 × M5 HiPer plus Taq HiFi PCR mix (with blue dye) (Mei5 Biotechnology Co., Ltd, Beijing, China), as recommended. PCR products were examined on 1% agarose gel by electrophoresis at 120 V for 15 min.

For the PCR using cDNA (qPCR) to verify the success expression of the foreign gene in transformants, a pair of gene specific primer CrtYqF3 (5′—GCACGCCACCATCCAGCAGTTCG—3′) and CrtYqR3 (5′—GCAAGCGGTCCGGGAGTGTCAGC—3′) targeting *CrtY* was designed. As an internal control, the expression of β-actin was also tested using the primer set Actin-F (5′—ACCCCGTGCTGCTGACTG—3′) and Actin-R (5′-ACGTTGAAGGTCTCGAACA—3′). The qPCR was performed on ABI 7300 Real-time PCR System (Applied Biosystems, Foster City, USA), in a 20 μL volume containing 1 μL cDNA, 10 μL 2 × SYBR^®^Premix Ex Taq TM II (Takara, Dalian, China), 0.8 μL each primer (10 μM), 0.4 μL ROX dye, and 7 μL nuclease-free ddH_2_O. The amplification condition was 98 ℃ for 15 s, 40 cycles of 98 ℃ for 10 s and 60 ℃ for 30 s. The Ct value was collected based on the default setting and 2^−ΔΔCT^ method was used to calculate the relative expression level of target gene. All qPCRs were performed at least in triplicates.

### Carotenoids extraction, identification and quantification

To extract carotenoids from *E. coli* and algal cells, harvested cells were dried using a freeze-dryer for 24 h. The carotenoids extraction, identification, and quantification in *E. coli* were processed as described previously [27]. The carotenoids determination in algal cells was described as following. The dried pellet of algal cells was crushed into powder, and then 10 mg were weight out and transferred into a 10 ml centrifuge tube. After adding 3 mL of cold methanol, cells were sonicated to release carotenoids. The supernatant was collected and filtered into a brown centrifuge tube and stored at − 20 °C for further analysis. Since there are non-target pigments presented in the extracts form algal cells, such as chlorophylls and lutein, a different HPLC detection procedure was applied, using the YMC C30 carotenoid column (4.6 × 250 mm, 5 micron). The HPLC conditions included the flow rate of 1 mL/min, the column temperature of 35 °C, the detection at 450 nm, and the injection volume of 10 μL. The mobile phase consisted of solution A (1% v/v phosphoric acid), solution B (tertiary-methyl-butyl ether), and solution C (methanol). Solution A was maintained at 4% during the whole gradient and the elution was initialed by 15% B and 81% C. The linear gradient included 20% B and 76% C at 2 min, 28% B and 68% C at 2.1 min, 28% B and 68% C at 13 min, 50% B and 46% C at 13.1 min, 70% B and 26% C at 18 min, 15% B and 81% C at 18.1 min, and 15% B and 81% C at 25 min. For the quantification, a calibration curve with coefficiency at higher than 0.99 was constructed using commercial standards, including lutein (HPLC ≥ 90%) and β-carotene (HPLC ≥ 90%) from Shanghai Yuanye Bio-Technology Co., Ltd. (Shanghai, China).

### Growth curve construction

To determine the growth of transgenic microalgae, the OD_750_ was measured in a period of 6 days. The seed culture at the OD_750_ of 0.3–0.4 was used for inoculation and three biological replicates were set up. The algal seed cells were diluted by TAP liquid medium into a final OD_750_ of 0.01, followed by the measurement of OD_750_ every day until Day 6. The measurement was performed on Epoch2 microplate reader (Bio-Tek Instruments, Winooski, VT, USA).

### Statistical analysis

All experiments were performed with at least three biological replicates. Data were presented as the mean with standard deviation. The statistical significance between two means was determined by Student’s t-test.

## Results

### Comparison of alga- and bacterium-driven lycopene β-cyclase in *E. coli*

**Fig. 2:**
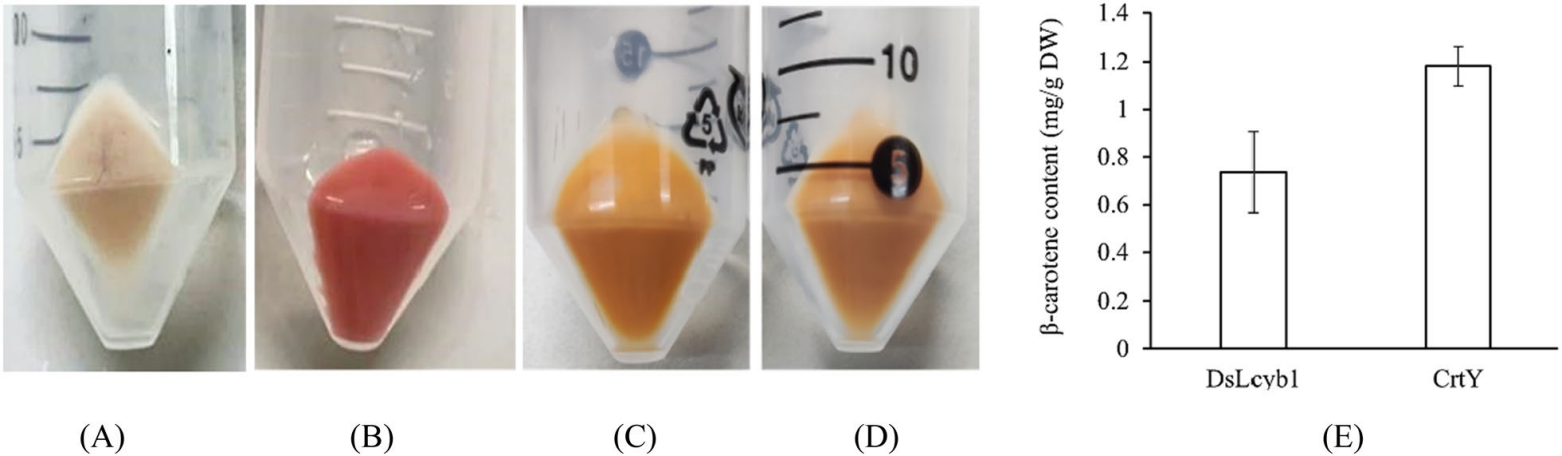
The detection and quantification of β-carotene productivity in *E*. *coli* by HPLC analysis. **A** The cell pellet of *E. coli* BL21(DE3); **B** the cell pellet of lycopene-produced *E*. *coli*; **C** the cell pellet of lycopene-produced *E*. *coli* with plasmid pET-DsLcyb1; **D** the cell pellet of lycopene-produced *E*. *coli* with plasmid pET-CrtY; **E** the amount of β-carotene in lycopene-produced *E*. *coli* with plasmid pET-DsLcyb1 or pET-CrtY

To compare the lycopene β-cyclase driven from bacterium and *Dunaliella salina* on β-carotene production, their performance was investigated in *E. coli*. After cultivation and induction, the *E. coli* cells BL21(DE3) without any additional foreigner genes had no additional color (Fig. 2A) and the *E. coli* cells producing lycopene displayed a red color (Fig. 2B). It is observed that the cell pellet of lycopene-produced E. coli containing either *DsLcyb1* or *CrtY* showed the yellow color (Fig. 2C and Fig. 2D), indicating that the lycopene was converted into other carotenoids with yellow color. Further pigment analysis using HPLC revealed that lycopene-produced *E. coli* transformed with alga-driven *DsLcyb1* or bacterium-driven *CrtY* generated β-carotene, at the level of 0.74 and 1.18 mg/g DW (dry weight), respectively (Fig. 2E). Statistically, *CrtY* performed better and produced appreciate 1.59 times of β-carotene than *DsLcyb1.*

### Nuclear transformation of *C. reinhardtii* and confirmation of transformants

**Fig. 3:**
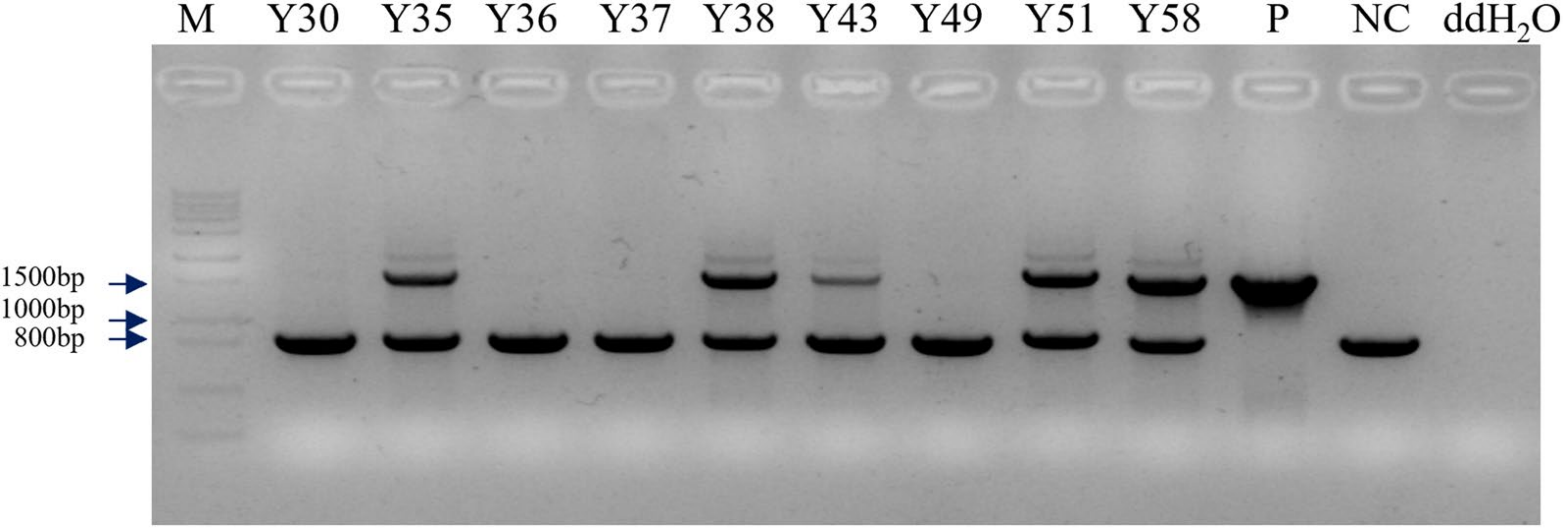
Identification of positive transformants using genomic DNA with the primer set psaD-P/psaD-T. M refers to the DNA ladder; P refers to the plasmid DNA of pDb124-CrtY, as the positive control; NC refers to the wild type of *C. reinhardtii*, as the negative control; ddH_2_O refers to the double distilled water, as the no template control; Y30, Y35, Y36, Y37, Y38, Y43,Y49, Y51, and Y58 refer to the algal colonies

To figure out if the motivated *CrtY* found in *E. coli* could promote the β-carotene biosynthesis in microalgae, the codon-optimized *CrtY* was transferred into *C. reinhardtii* genome. The PCR was performed to evaluate the successful integration of *CrtY* into *C. reinhardtii* nuclear genome, using genomic DNA isolated from colonies grown under antibiotic selection stress. According to the feature of primer set psaD-P/psaD-T which will amplify *CrtY* gene as well as the native *psaD* gene in the *C. reinhardtii* genome, the positive transformants should generate two PCR fragments, including the 813 bp of native *psaD* gene and the 1590 bp of *CrtY* gene. Results suggested that the negative control (NC, the wild type of *C. reinhardtii*) and the positive control (P, the plasmid DNA of pDb124-CrtY) generated PCR fragments in expected size, indicating the good performance of psaD-P/psaD-T in the PCR (Fig. 3). As a result, algal colonies Y35, Y38, Y43, Y51, and Y58 generated two fragments that were in the same size as NC and P, suggesting the successful integration of *CrtY* in their genome.

**Fig. 4:**
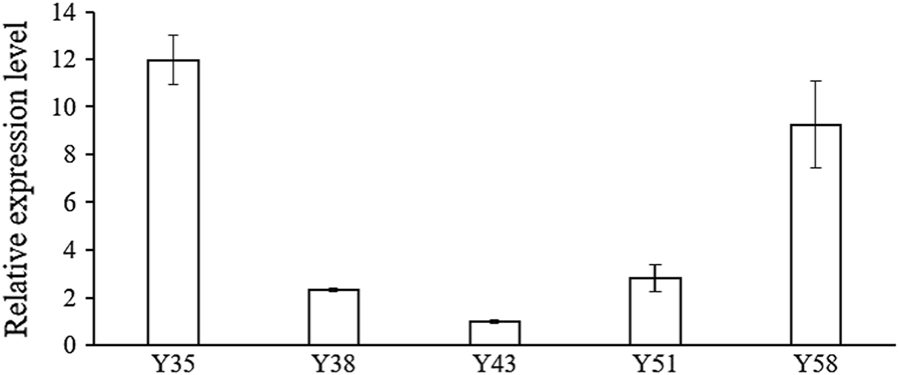
The relative expression levels of *C. reinhardtii* transformants based on qPCR analysis. The expression level of actin was set as the internal control and the expression level of *CrtY* in Y43 was used for data normalization

Subsequently, to confirm the successful expression of *CrtY* in positive transformants of *C. reinhardtii*, the qPCR was conducted. The primer set CrtYqF3/ CrtYqR3 was specific and located within the coding region of *CrtY*, no amplification was observed in the wild type of *C. reinhardtii*. Hence, to normalize the relative expression level of *CrtY* in transformants, the transformant Y43 was employed as the control. Results suggested that there were significant differences on the relative expression level of *CrtY* among transformants (Fig. 4). The high expression level of *CrtY* was found in Y35 and Y58, which is about 12.0 and 9.2 times of that in Y43. Transformants Y38 and Y51 had similar transcripts of *CrtY*, that were about 2.3 and 2.8 times of that in Y43, respectively.

### Carotenoids profile and growth of *C. reinhardtii* transformants

**Fig. 5:**
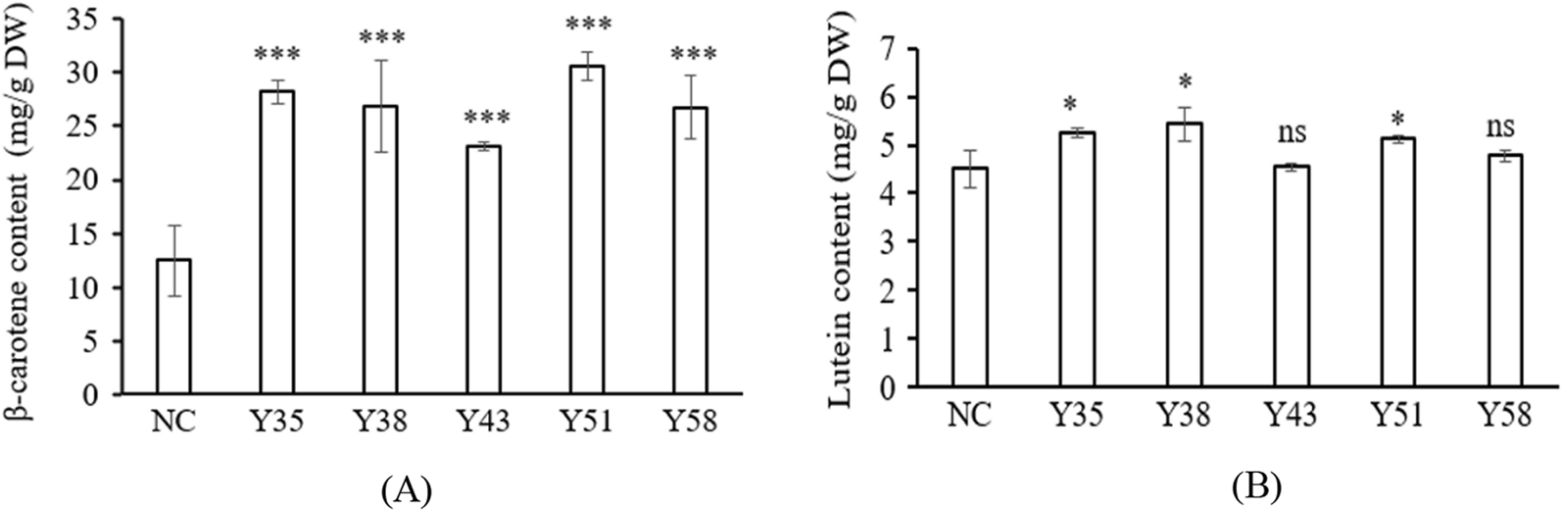
The amount of β-carotene (**A**) and lutein (**B**) in the wild type and transgenic *C. reinhardtii* based on the HPLC analysis. NC indicates the wild type of *C. reinhardtii*, as the negative control. Y35, Y38, Y43, Y51, and Y58 indicate different strains of transgenic *C. reinhardtii*. * and *** indicate the statistical significance at the level of 0.05 and 0.001, respectively. ns indicates no statistical significance at the level of 0.05

To determine the effect of expressing *CrtY* in the transgenic *C. reinhardtii* on carotenoids biosynthesis, pigments were extracted from algal cells suffering from the high light treatment which has been reported as a stimulation to promote carotenoids biosynthesis. The amounts of β-carotene and lutein are expected to increase and reduce in transgenic algal cells, respectively. Based on the HPLC analysis, the β-carotene content in transgenic *C. reinhardtii* was varied from 23.13 to 30.65 mg/g DW, while that in wild type of *C. reinhardtii* was 12.48 mg/g. Therefore, all tested transgenic *C. reinhardtii* produced significant higher amount of β-carotene than the wild type (Fig. 5A), at the confidence of 0.001. The highest amount of β-carotene was found in Y51, which had 2.45 times of β-carotene than the wild type. Compared with β-carotene, the lutein content was much lower, ranging from 4.50 to 5.43 mg/g DW. Statistical analysis revealed that only Y35, Y38, and Y51 had significant higher amount of lutein than the wild type, at the confidence of 0.05, while Y43 and Y58 had similar amount of lutein as the wild type (Fig. 5B). By comparing the data, the highest lutein production was found in Y38, which was only 1.2 times of that in the wild type.

**Fig. 6:**
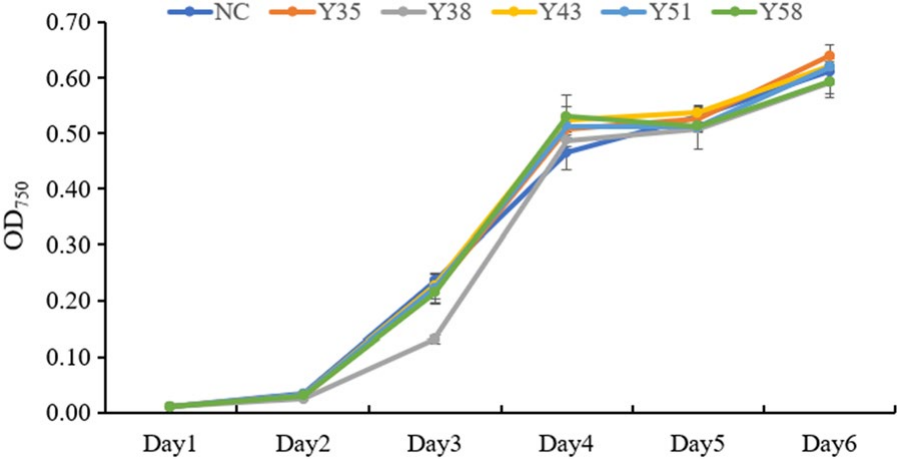
The growth curve of the wild type and transgenic *C. reinhardtii.* NC stands for the wild type of *C. reinhardtii*, as the negative control. Y35, Y38, Y43, Y51, and Y58 refer to different strains of transgenic *C. reinhardtii*

Moreover, to check if the overexpression of *CrtY* affects the growth of microalgae, the growth curve of transgenic *C. reinhardtii* was measured. Results suggested that except Y38, which showed obvious growth defect at Day3, all other transgenic *C. reinhardtii* showed no difference on the growth with the wild type at all determined points (Fig. 6). Moreover, the growth of Y38 was recovered at Day 4 and maintained similar growth status as the wild type. Hence, it was concluded that the overexpression of *CrtY* had no defective effect on the microalga growth.

## Discussion

β-Carotene is a terpenoid with high economic value [7]. With the advantage of well-elucidated pathway of β-carotene biosynthesis, lots of efforts were placed to bio-engineer its production with high yield and low cost, mainly using *E. coli* and yeast as cell factory [8–10]. However, only few researches on the production of β-carotene in microalgae were reported, even though microalgae were considered as the green cell factory for drug-related products, due to the advantages of low cultivation cost, fast growth, and fixation of CO_2_ as the carbon source [28]. Aiming to construct a microalgae strain with high β-carotene productively, a set of experiments was designed in this study, using microalgae *Chlamydomonas reinhardtii* as the platform.

As documented, the biosynthesis of β-carotene starts from IPP (isopentenyl pyrophosphate), followed by various enzymes including isopentenyl pyrophosphate isomerase, geranylgeranyl diphosphate synthase, phytoene desaturase, phytoene synthase, and lycopene cyclase, the C5 blocks were built into C40 carotene [1]. The lycopene cyclase [EC: 5.5.1.19] which cyclize the linear C40-lycopene and introduce the β- or ε-ionone end groups leads to the formation of β-carotene (containing only β-rings) and α‐carotene (containing one β-ring and one ε-ring) [1–3]. Four families of lycopene cyclase have been reported and their similarities with each other are low [19–21]. Among them, lycopene β-cyclase introduces one β-ring at one end of lycopene to form γ-carotene, and then introduces another β-ring to the other end to form β-carotene [21]. The lycopene β-cyclases from carotenogenic bacteria (CrtY) and from plants, algae, and cyanobacteria (CrtL-b and LCY-b) were proved to have good activity for carotenoids production in plants and engineered bacteria [21]. In microalgae, it has been reported that the microalga *D. salina* accumulated the highest amount of natural β-carotene, which is about 10% of the dry weight, leading to the possibility that *DsLcyb1* might have superior activity for β-carotene biosynthesis [29, 30]. Besides, the bacterial *CrtY* has been frequently used to produce β-carotene in *E. coli* [23]. Therefore, this study selected *CrtY* from bacteria *Pantoea agglomerans* and LCY-b from microalga *Dunaliella salina* (*DsLcyb1*) as candidates, aiming to improve the β-carotene production in *C. reinhardtii* by overexpressing an effective lycopene cyclase. According to the results obtained in this study, it was found that *CrtY* has better activity than *DsLcyb1* on β-carotene production in *E. coli* (Fig. 2). Furthermore, the bacterial *CrtY* was attempt to integrate into the nuclear genome of *C. reinhardtii*, with the expect to competitively and dominantly convert lycopene into β-carotene rather than α-carotene. Thereby, the transgenic *C. reinhardtii* was expected to have increased β-carotene and reduced lutein content. It turns out the overexpression of *CrtY* dramatically increased the β-carotene from 12.48 mg/g DW to 30.65 mg/g DW, which is about 2.45-fold increment (Fig. 5). However, the reduction of lutein was not observed. By contrast, the lutein content was even slightly increased in some transformants (Fig. 5). It was also found that the transgenic microalgae were grew as well as the wild type (Fig. 6). This finding suggested that the increment of β-carotene was not due to the reduced carotenoids flux into α-carotene, leading to the fact that there was no tradeoff between α-carotene and β-carotene when overexpressing lycopene β-cyclase in *C. reinhardtii*. It is noted that the expression levels of *CrtY* in transgenic microalgal strains are not exactly consistent with the β-carotene content, since the highest β-carotene content was found in Y51(Fig. 5A), while the most abundant *CrtY* transcripts were found in Y35 (Fig. 4). Previously, in a study overexpressing native *CrLCYE* in *C. reinhardtii* aiming to improve lutein productivity, the transformants CrCLYE#L6 had the significant lower transcripts but had similar increased lutein content as other transformants [22], implying that post-transcription regulation should be involved during down-stream carotenoids biosynthesis.

In *C. reinhardtii*, several researches have been conducted to genetically manipulate carotenoids biosynthesis, mainly including the overexpression of endogenous genes and foreigner genes from *Chlorella zofingiensis*, *D. salina, Haematococcus pluvialis*, and *Xanthophyllomyces dendrorhous* to promote carotenoids production [31, 32]. Without exception, no bacteria-driven genes that were widely used in *E. coli* for carotenoids production were attempted to be used in *C. reinhardtii* or other microalgae. Commonly, gene expression and its regulation in eukaryotic cells are much complex than in prokaryotic cells [33], leading to the suspicion that the superior gene tested by *E. coli* can also perform well in eukaryotic cells. No references clearly point out the answer. This study firstly overexpressed a bacterial gene *CrtY* encoding an enzyme with the function to convert lycopene into β-carotene in *C. reinhardtii*. This gene was well functioned which was evidenced by dramatically increased β-carotene content in transgenic *C. reinhardtii*. The results in this study will widen the gene cluster for carotenoids biofortification in microalgae.

### Supplementary Information

**Additional file 1: Figure S1.** Schematic map of pAC-Lyco and pDb124 used in this study.

## Data Availability

The datasets supporting the conclusions of this article are included within the article or the additional file (Additional file 1: Fig. S1).
