# Supplementary figures and images for: Enhancement of β-carotene content in Chlamydomonas reinhardtii by expressing bacterium-driven lycopene β-cyclase
Source: Biotechnol Biofuels Bioprod. 2023 Aug 12;16:127. doi: 10.1186/s13068-023-02377-1 (PMC10423417; doi:10.1186/s13068-023-02377-1)

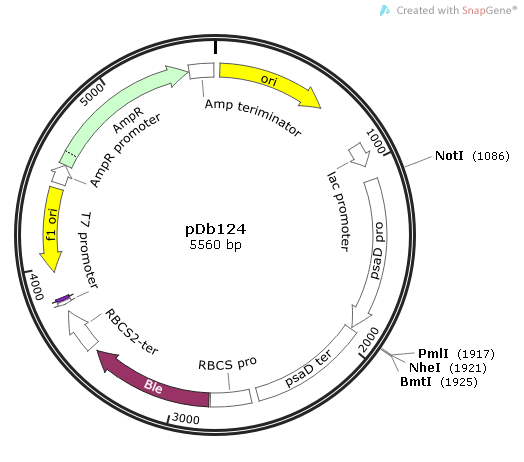

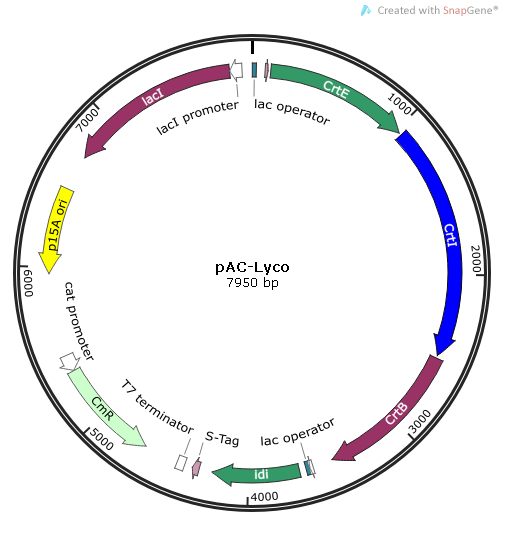


1. (B)

**Fig. S1** Schematic map of pAC-Lyco and pDb124 used in this study.

Supplement: Supplementary file 1 — Additional file 1: Figure S1. Schematic map of pAC-Lyco and pDb124 used in this study. [file 13068_2023_2377_MOESM1_ESM.docx]
